# Meta-analysis on the efficacy and safety of rituximab versus tacrolimus for nephrotic syndrome in the paediatric age group

**DOI:** 10.1093/ckj/sfad263

**Published:** 2023-10-13

**Authors:** Syeda Tayyaba Rehan, Eman Ali, Farea Eqbal, Muhammad Nadeem Ahsan, Muhammad Sohaib Asghar

**Affiliations:** Dow University of Health Sciences, Karachi, Pakistan; Dow University of Health Sciences, Karachi, Pakistan; Dow University of Health Sciences, Karachi, Pakistan; Dow University of Health Sciences, Karachi, Pakistan; Mayo Clinic Rochester, MN, USA

To the Editor,

Nephrotic syndrome (NS), characterized by a triad of massive proteinuria, generalized oedema and hypoalbuminemia, is a leading cause of chronic kidney disease (CKD), affecting 1.15–16.9 per 100 000 children per year [1]. Over the past few decades, corticosteroids, particularly prednisolone, have been the mainstay of treatment for NS among children [1]. Approximately 40% of patients experience frequent relapses and are known to be steroid-dependent [2]. Calcineurin inhibitor, tacrolimus and rituximab, an immunosuppressive agent, are other valuable treatment options for managing children with steroid-dependent NS [2–4]. A scarcity of studies comparing the safety and efficacy of potential therapeutic options and the inconsistency among the findings of existing studies make it vital to pool the results in order to reach a conclusion regarding the definitive treatment plan for NS. Therefore, in this meta-analysis we aimed to compare the efficacy, safety and success of rituximab and tacrolimus among paediatric patients with steroid-sensitive and steroid-dependent NS.

We performed a comprehensive electronic search (Supplementary Table S1) through June 2023, including all randomized control trials (RCTs) and cohort studies that compared rituximab with tacrolimus among the paediatric population (<18 years of age) with corticosteroid-dependent or steroid-sensitive NS. The primary endpoints evaluated were relapse-free survival rate, cumulative prednisolone dosage, treatment failure and number of patients with adverse effects. Changes in serum biomarkers from baseline, including albumin, cholesterol and estimated glomerular filtration rate (eGFR) were the secondary outcomes. All the endpoints were evaluated at the 1-year follow-up. ReviewManager (version 5.3; Cochrane Collaboration, London, UK) was employed for the pooled statistical analysis in this article. *P*-values <.05 were considered significant throughout the article. The risk of bias in the included studies was assessed using the Newcastle–Ottawa scale for cohort studies and the Cochrane bias assessment tool for RCTs.

Two RCTs [2, 3] and one cohort study [4] were included in this article. Of the total 184 patients, 91 belonged to the rituximab group while 93 patients were administered tacrolimus. Baseline characteristics are highlighted in Supplementary Table S2. Statistical analysis demonstrated a significantly lower risk of relapse among the rituximab group as compared with tacrolimus {relative risk [RR] 0.35 [95% confidence interval (CI) 1.11–1.63]; *P* = .002; *I*^2^ = 0%} (Supplementary Fig. S2A). An insignificant reduction in cumulative prednisolone dosage between the two comparable groups [weighted mean difference (WMD) = −21.88 (95% CI −67.50–23.74); *P* = 0.35; *I*^2^ = 68%] and no significant risk of treatment failure [RR 1.11 (95% CI 0.28–4.42); *P* = 0.88; *I*^2^ = 65%] and developing adverse events was noted with rituximab as compared with tacrolimus [RR 1.11 (95% CI 0.28–4.42); *P* = .88; *I*^2^ = 65%] (Supplementary Figs. S2B–D). A non-significant reduction in the serum albumin, increase in serum cholesterol levels [WMD = 27.12 (95% CI −38.40–92.65); *P* = .42; *I*^2^ = 87%] and eGFR [WMD = 6.36 (95% CI −4.88–17.61); *P* = .27; *I*^2^ = 95%] was noted at the 1-year follow-up among the two comparable groups [WMD = −0.15 (95% CI −0.80–1.10); *P* = .76; *I*^2^ = 96%] (Supplementary Figs. S3A–C). Of the three included studies, two [2, 4] were reported to have good quality, whereas one RCT [3] raised some concerns due to possible deviation from the intended interventions (Supplementary Table S3, Figs. S4 and S5).

Contrary to the results of the previous analysis [5], our study revealed promising results in terms of relapse-free survival for rituximab. Furthermore, Sinha *et al.* [6] found that sequential therapy with rituximab effectively reduces relapses in patients with steroid-dependent or refractory NS, however, therapy is associated with high rates of hypogammaglobulinaemia and infusion reactions. Small sample size hinders the credibility of our findings, but the absence of in-study heterogeneity for this outcome enhances its authenticity and impact. As for the remaining endpoints, including the risk of developing adverse events and improving the levels of serum markers, no evident differences were illustrated between the rituximab and tacrolimus groups. The methodological variation among the selected studies, as well as inconsistency in the severity and steroid dependence of NS among the patient population are some of the factors that could contribute to heterogeneity. Therefore, large-sample, multicentre studies are needed in order to reach a valid conclusion regarding safe and efficacious treatment options for steroid-dependent NS.

## Supplementary Material

sfad263_Supplemental_File
