# Supplementary material for: Meta-analysis on the efficacy and safety of rituximab versus tacrolimus for nephrotic syndrome in the paediatric age group
Source: Clin Kidney J. 2023 Oct 13;17(1):sfad263. doi: 10.1093/ckj/sfad263 (PMC10768748; doi:10.1093/ckj/sfad263)
Supplement: sfad263_Supplemental_File [file sfad263_supplemental_file.docx]

**Electronic Supplementary Material**

**Table S1:** Search strategy used in each database searched.

| Database | Search Strategy | Articles retrieved |
| --- | --- | --- |
| PubMed | ("rituximab"[MeSH Terms] OR "rituximab"[All Fields] OR "rituximab s"[All Fields]) AND ("tacrolimus"[MeSH Terms] OR "tacrolimus"[All Fields]) AND ("nephrotic syndrome"[MeSH Terms] OR ("nephrotic"[All Fields] AND "syndrome"[All Fields]) OR "nephrotic syndrome"[All Fields] OR ("steroid"[All Fields] AND "dependent"[All Fields] AND "nephrotic"[All Fields] AND "syndrome"[All Fields]) OR "steroid dependent nephrotic syndrome"[All Fields] OR ("nephrotic syndrome"[MeSH Terms] OR ("nephrotic"[All Fields] AND "syndrome"[All Fields]) OR "nephrotic syndrome"[All Fields] OR ("steroid"[All Fields] AND "sensitive"[All Fields] AND "nephrotic"[All Fields] AND "syndrome"[All Fields]) OR "steroid sensitive nephrotic syndrome"[All Fields])) | 82 |
| Cochrane Library | (Rituximab) AND (Tacrolimus) AND (Steroid-dependent Nephrotic Syndrome OR steroid-sensitive nephrotic syndrome) | 15 |
| Google Scholar | (Rituximab) AND (Tacrolimus) AND (Steroid-dependent Nephrotic Syndrome OR steroid-sensitive nephrotic syndrome) | 1280 |
| ScienceDirect | (Rituximab) AND (Tacrolimus) AND (Steroid-dependent Nephrotic Syndrome OR steroid-sensitive nephrotic syndrome) | 620 |
| ClinicalTrials.Gov | (Rituximab) AND (Tacrolimus) AND (Steroid-dependent Nephrotic Syndrome) | 1 |

**Table S2: Baseline for included studies.**

|  | **Demographics** | | | | | | **Anthropometry** | | | | **Serum biochemistry** | | | | | |
| --- | --- | --- | --- | --- | --- | --- | --- | --- | --- | --- | --- | --- | --- | --- | --- | --- |
|  | **Sample size** | | **Male No. (%)** | | **Age (Mean ± SD)** | | **Height (Z-score)** | | **BMI (Z-score)** | | **Albumin, g/dl** | | **Cholesterol, mg/dl** | | **Estimated GFR, ml/min/1.73 m2** | |
| **Author, year** | **Rituximab** | **Tacrolimus** | **Rituximab** | **Tacrolimus** | **Rituximab** | **Tacrolimus** | **Rituximab** | **Tacrolimus** | **Rituximab** | **Tacrolimus** | **Rituximab** | **Tacrolimus** | **Rituximab** | **Tacrolimus** | **Rituximab** | **Tacrolimus** |
| Sinha 2011 | 10 | 13 | 8 (80%) | 10 (76.9%) | 12.2 ± 2.3 | 12.3 ± 3.0 | −2.2 ± 1.1 | −2.3 ± 1.8 | 2.0 ± 1.1 | 1.7 ± 0.8 | 3.8 ± 0.5 | 3.1 ± 0.9 | 221.3 ± 40.2 | 295.8 ± 151.1 | 102.6 ± 28.3 | 96.7 ± 25.8 |
| Basu 2018 | 60 | 60 | 32 (53.3) | 32 (53.3) | 7.1 ± 2.8 | 7.2 ± 2.8 | −1.4 ± 0.7 | −1.2 ± 0.6 | 2.2 ± 1.0 | 2.2 (0.9) | 4.18 ± 0.73 | 4.34 ± 0.81 | 109 ± 23 | 115 ± 24 | 100.2 ± 8.6 | 103.0 ± 10.8 |
| Mathew 2022 | 21 | 20 | 15 (71.4) | 19 (95) | 8.99 ± 2.98 | 10.5 ± 5.5 | -1.21 ± 0.87 | –1.46 ± 0.83 | 1.18 ± 0.96 | 0.66 ± 1.29 | 3.8 ± 0.56 | 3.93 ± 0.72 | - | - | 138.2 ± 22.2 | 139.4 ± 25.9 |

**Table S3:** Quality assessment of observational studies using Newcastle Ottawa Scale

| Study/Score | Selection | | | | Comparability | | Outcome | | |  |
| --- | --- | --- | --- | --- | --- | --- | --- | --- | --- | --- |
|  | S1 | S2 | S3 | S4 | C |  | O1 | O2 | O3 | Total |
| Sinha et al. 2011 | * | * | * |  | * |  | * | * | * | 7 |


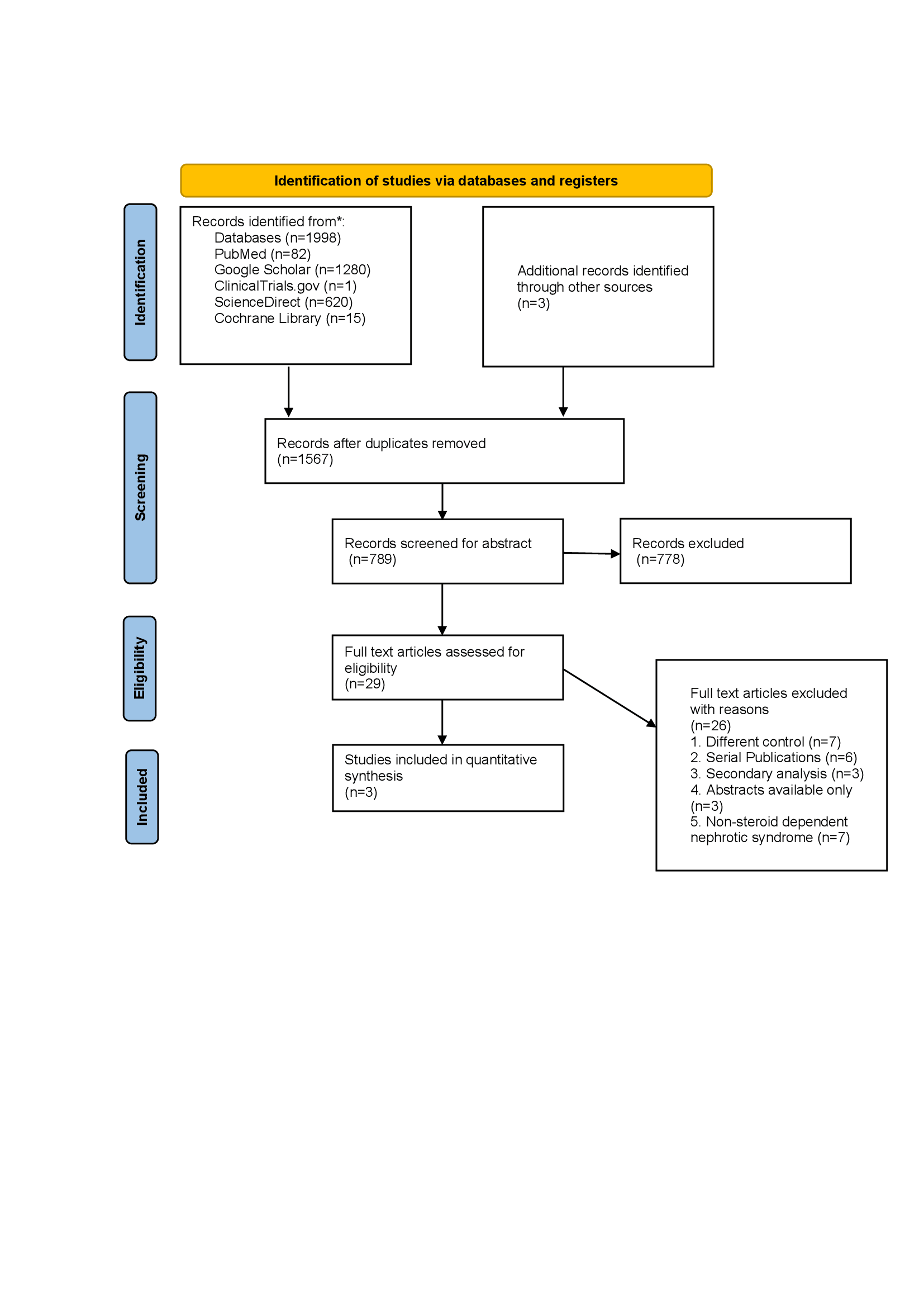


**Figure S1**: PRISMA flow diagram


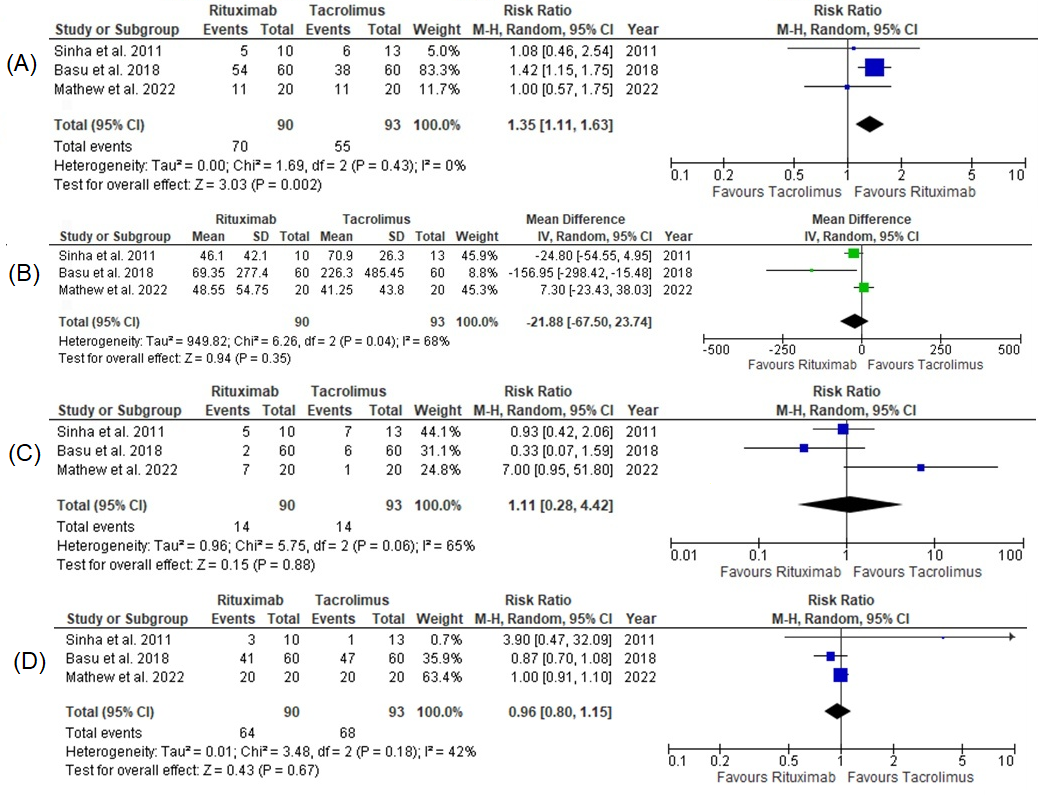


**Figure S2:** Forest plots depicting effect sizes for A) Relapse-free survival rate at 1-Year follow-up B) Cumulative prednisolone dosage (mg/kg/year) at 1-year follow-up C) Treatment Failure at 1-year follow-up D) No. of patients with adverse effects at 1-year follow-up.


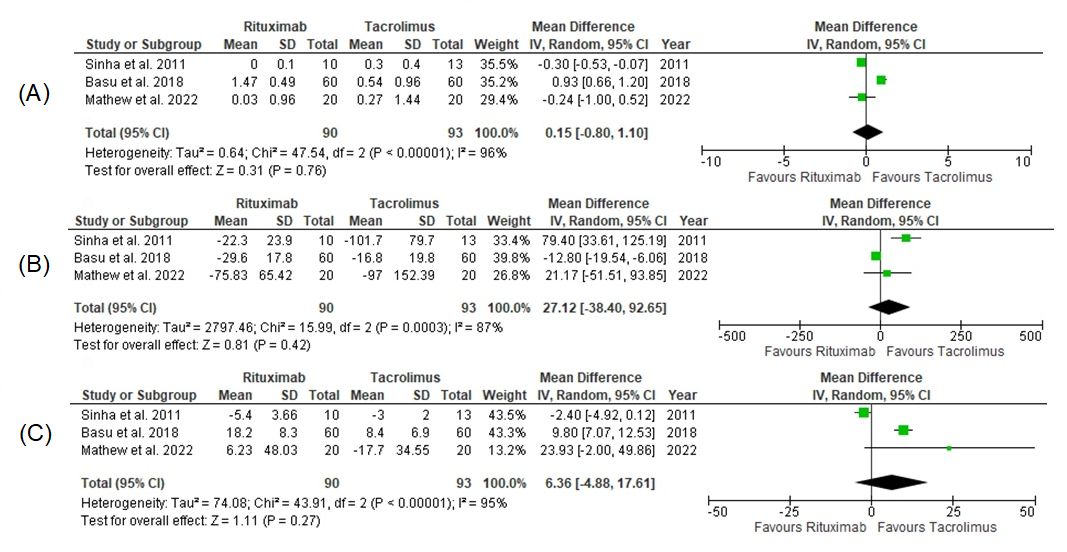


**Figure S3:** Forest plots depicting effect sizes for A) Changes in serum albumin levels (g/dl) at 1-Year follow-up B) Changes in serum cholesterol (mg/dl) levels at 1-year follow-up C) Changes in eGFR (ml/min/1.73m^2^) at 1-Year follow up.


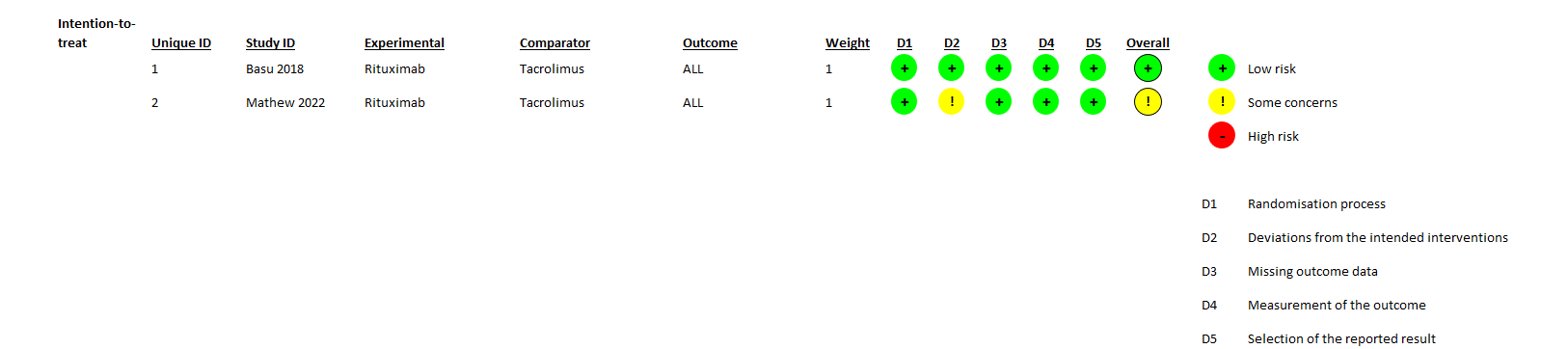


**Figure S4:**  ROB-2 analysis of included RCTs.

**Figure S5**: Risk of bias graph: review authors’ judgments about each risk of bias item presented as percentages across all included studies.
